# Conservative Approach versus Percutaneous Coronary Intervention in Patients with Spontaneous Coronary Artery Dissection from a National Population-Based Cohort Study

**DOI:** 10.31083/j.rcm2511404

**Published:** 2024-11-18

**Authors:** Chayakrit Krittanawong, Beatriz Castillo Rodriguez, Song Peng Ang, Yusuf Kamran Qadeer, Zhen Wang, Mahboob Alam, Samin Sharma, Hani Jneid

**Affiliations:** ^1^Cardiology Division, NYU Langone Health and NYU School of Medicine, New York, NY 10016, USA; ^2^Division of Internal Medicine, Baylor College of Medicine, Houston, TX 77030, USA; ^3^Division of Internal Medicine, Rutgers Health Community Medical Center, Toms River, NJ 08755, USA; ^4^Division of Cardiology, Department of Medicine, Henry Ford Hospital, Detroit, MI 48202, USA; ^5^Robert D. and Patricia E. Kern Center for the Science of Health Care Delivery, Mayo Clinic, Rochester, MN 55902, USA; ^6^Division of Health Care Policy and Research, Department of Health Sciences Research, Mayo Clinic, Rochester, MN 55902, USA; ^7^The Texas Heart Institute, Baylor College of Medicine, Houston, TX 77030, USA; ^8^Cardiac Catheterization Laboratory of the Cardiovascular Institute, Mount Sinai Hospital, New York, NY 10029, USA; ^9^Divisionof Cardiology, University of Texas Medical Branch, Houston, TX 77555, USA

**Keywords:** spontaneous coronary artery dissection, PCI, acute coronary syndrome

## Abstract

**Background:**

Spontaneous coronary artery dissection (SCAD) is a rare and often underdiagnosed cause of acute coronary syndrome (ACS), predominantly affecting younger women without traditional cardiovascular risk factors. The management of SCAD remains a subject of debate, likely secondary to inconclusive evidence. This study aims to compare the clinical outcomes of SCAD patients treated with optimal medical therapy (OMT) versus those who underwent percutaneous coronary intervention (PCI) using a national population-based cohort.

**Methods:**

We conducted a retrospective analysis using the National Inpatient Sample (NIS) database from 2016 to 2020. The study included patients identified with SCAD using the ICD-10-CM (the International Classification of Diseases, Tenth Revision, Clinical Modification) code I25.42. We excluded individuals who did not receive PCI or coronary angiography, those who underwent coronary artery bypass grafting, and patients with incomplete records. The primary outcome was in-hospital mortality, while secondary outcomes included acute kidney injury, cardiac arrest, cardiogenic shock, use of temporary mechanical circulatory support, cost of hospitalization, and length of stay. National estimates were obtained using discharge weights, and statistical comparisons were performed using chi-square tests and linear regression. Multivariate logistic regression was employed to identify predictors of mortality and other outcomes.

**Results:**

A total of 31,105 SCAD patients were included in the study, with 10,480 receiving OMT and 20,625 undergoing PCI. Patients in the PCI group were older (mean age 64 vs. 54 years) and had higher comorbidities compared to those in the OMT group. The proportion of SCAD patients receiving PCI declined from 72% in 2016 to 60% in 2020. In multivariable analysis, PCI was associated with increased in-hospital mortality (odds ratio (OR) 1.89, 95% confidence interval (CI) 1.24–2.90, *p* = 0.0003), cardiogenic shock (OR 2.29, 95% CI 1.71–3.07, *p* < 0.0001), use of a left ventricular assist device (LVAD) (OR 3.97, 95% CI 2.42–6.53, *p* < 0.0001), and an intra-aortic balloon pump (IABP) (OR 2.24, 95% CI 1.63–3.09, *p* < 0.0001). Trends also suggested an association between PCI and cardiac arrest, extracorporeal membrane oxygenation (ECMO), and acute kidney injury (AKI). The PCI group had significantly higher hospitalization costs and longer lengths of stay compared to the OMT group (both *p* < 0.001).

**Conclusions:**

In this large, national cohort study, SCAD patients who underwent PCI had significantly higher risks of adverse in-hospital outcomes, including mortality, compared to those treated with OMT. These findings underscore the importance of careful patient selection and the potential advantages of conservative management in SCAD, particularly in patients without severe or unstable presentations. Further research is needed to develop evidence-based guidelines for the optimal management of SCAD.

## 1. Introduction

Spontaneous coronary artery dissection (SCAD) is a rare but increasingly recognized cause of acute coronary syndrome (ACS), accounting for a small yet significant proportion of ACS cases, particularly in younger women without traditional cardiovascular risk factors [1, 2, 3]. SCAD is characterized by the separation of the coronary artery wall layers, which leads to the formation of a false lumen and subsequent compromise of blood flow, potentially resulting in myocardial ischemia, infarction, and even sudden cardiac death. The incidence of SCAD is reported to be between 0.1% and 1.1% of all cases of ACS, though it is likely underdiagnosed due to its variable presentation and the challenges in detection using conventional coronary angiography. The pathophysiology of SCAD is distinct from atherosclerotic coronary artery disease, as it is not associated with plaque rupture or thrombosis. Instead, the dissection typically occurs within the intima or media of the coronary artery, creating an intramural hematoma that compresses the true lumen [4, 5, 6]. This unique mechanism of ischemia poses significant challenges in the management of SCAD, as traditional interventional strategies, such as percutaneous coronary intervention (PCI), which are effective in atherosclerotic ACS, but may not be appropriate or effective in SCAD.

The management of SCAD remains a subject of debate due to the lack of randomized controlled trials (RCTs) specifically addressing the optimal treatment strategy. The most commonly employed strategy is conservative management with optimal medical therapy (OMT), which may include antiplatelet agents, beta-blockers, and angiotensin-converting enzyme (ACE) inhibitors [7]. This approach is often preferred due to the potential for spontaneous healing of the dissection and the high risk of procedural complications associated with PCI in SCAD patients [8]. Despite the preference for conservative management, there are circumstances where revascularization may be considered, particularly in patients with ongoing ischemia, left main or proximal artery involvement, or hemodynamic instability. In such cases, the decision to pursue PCI must be made cautiously, weighing the risks of the procedure against the potential benefits.

Given the complexities and risks associated with SCAD management, there is a pressing need for more robust data to guide treatment decisions. To address this gap, we performed analyses using a national population-based cohort to evaluate the clinical outcomes of SCAD patients managed with OMT versus those who underwent PCI.

## 2. Methods

We performed a retrospective study using the National Inpatient Sample (NIS) database from 2016 to 2020. NIS is one of the largest national databases that contains information from approximately 7 million hospital stays annually in its unweighted form. When weighted, it could project up to 35 million hospitalizations across the nation each year. The data contained in this database is deidentified, thus, the approval from the Institutional Review Board (IRB) was not required.

### 2.1 Study Population

In this study, we identified hospital admissions for SCAD by using the ICD-10-CM (the International Classification of Diseases, Tenth Revision, Clinical Modification) code I25.42. In line with previous analyses concerning SCAD patient populations, we excluded individuals who did not receive PCI or coronary angiography to maintain diagnostic precision. Additionally, we excluded patients who underwent concurrent coronary artery bypass grafting and those with a diagnosis of accidental puncture to preserve the homogeneity of our study cohort. We also omitted data from patients with incomplete or missing records pertaining to age, gender, or mortality.

### 2.2 Outcomes

Our primary outcome was in-hospital mortality. Secondary outcomes included acute kidney injury, cardiac arrest, cardiogenic shock, use of temporary mechanical circulatory support, cost of hospitalization and length of stay.

### 2.3 Statistical Analysis

We obtained the national estimates using the discharge weight provided within the database. We described dichotomous variables using frequencies and/or percentages and compared them using the chi-square test. Non-dichotomous variables were described in mean and standard deviation and comparison was made using linear regression. Hospitalization trends were demonstrated using a bar chart. We described the raw unadjusted outcomes and subsequently performed multivariate logistic regression analysis, using variables that were significant on univariate analysis with a threshold of 0.05. To control for confounding variables and improve the comparability between treatment and control groups, we employed propensity score matching using the caliper method. We then performed nearest neighbor matching with a caliper width set to 0.2 of the standard deviation of the logit of the propensity score to reduce bias. In assessing the outcomes using the matched data, we constructed 2 models. Model 1 was analyzed using only the matched data, while Model 2 expanded upon Model 1 by additionally adjusting for covariates that remained significant in the univariate analysis. We further assessed the predictors of mortality among those who received PCI and those who received OMT respectively using the similar regression approach as described earlier. All statistical analyses were conducted using STATA version 17.0 (StataCorp, College Station, TX, USA).

## 3. Results

**Fig. 1.**
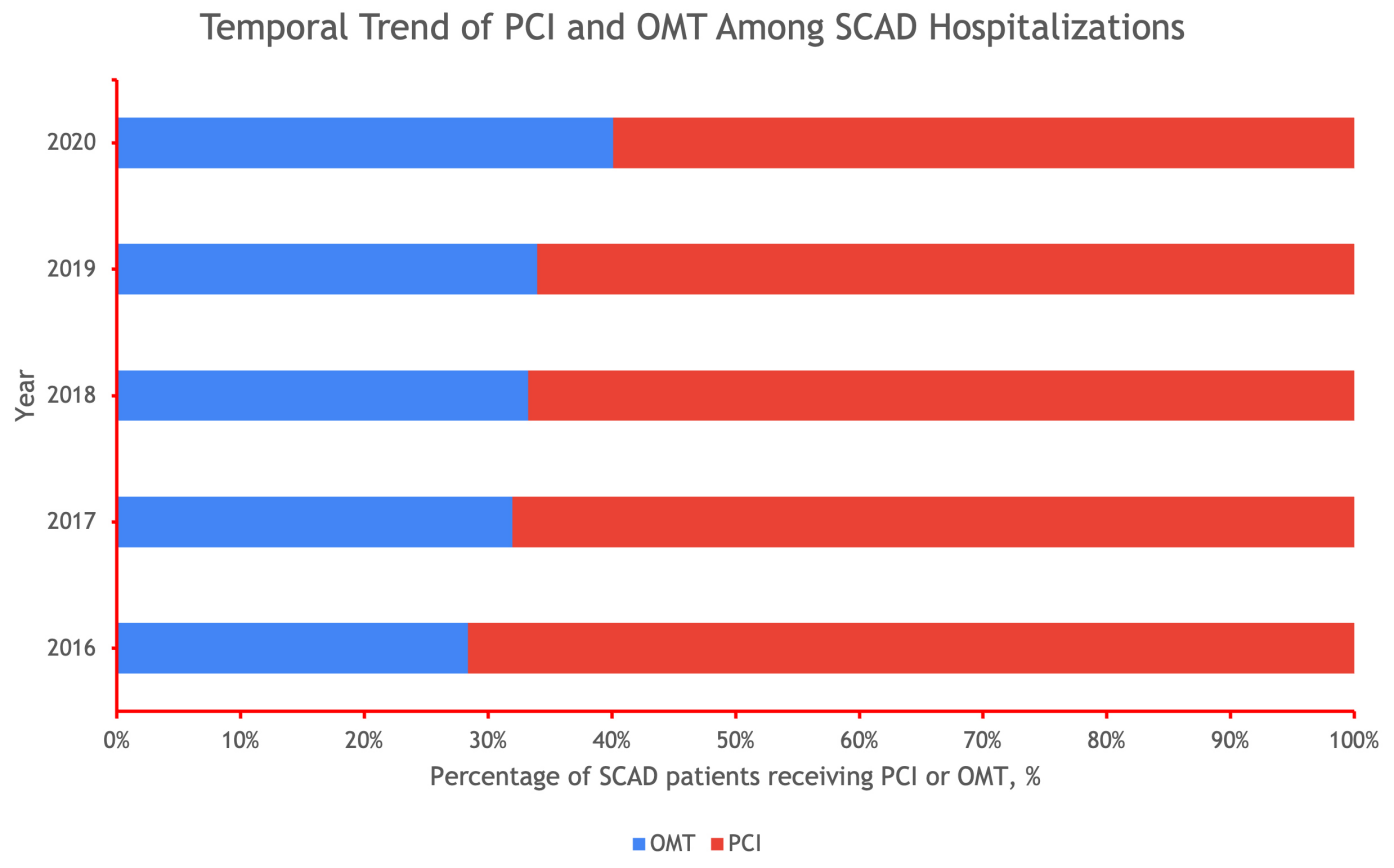
**Temporal Trend of Percutaneous Coronary Intervention (PCI) and Optimal Medical Therapy (OMT) Among Spontaneous Coronary Artery Dissection (SCAD) Hospitalizations**.

We analyzed 31,105 patients with SCAD, 10,480 of which received OMT and 20,625 of which had PCI. Of the patients with SCAD who had PCI, the mean age was 64 years with 48% of the patients being female and most patients being Caucasian. In comparison, of the patients with SCAD who received optimal medical therapy, the mean age was 54 years with 78% of the patients being female and most patients being Caucasian. In the SCAD receiving PCI compared to the SCAD receiving OMT, the SCAD receiving PCI population had significantly higher comorbidities including cardiac arrhythmias, congestive heart failure, valvular heart disorders, peripheral valvular disease, hypertension, chronic lung disease, diabetes mellitus, fluid disorders, chronic kidney disease (CKD), smoking, and prior stroke (Table 1). The rate of PCI has declined yearly from 72% in 2016 to 60% in 2020 (Fig. 1).

**Table 1.** **Baseline characteristics of SCAD patients between OMT versus PCI**.

Variables		OMT		PCI		Total	*p*-value
		n	%	n	%		
Number of patients, n		10,480		20,625		31,105	
Age		54.37 ± 13.61		64.01 ± 13.64			<0.001
Female		8195	78.20	9920	48.10	18,115	<0.001
Race							<0.001
	White	6985	66.65	15,095	73.19	22,080	
	Black	1550	14.79	1820	8.82	3370	
	Hispanic	1025	9.78	1650	8.00	2675	
	Asian or Pacific Islander	220	2.10	470	2.28	690	
	Native American	40	0.38	90	0.44	130	
	Other	225	2.15	555	2.69	780	
Hospital bed size							0.024
	Small	1375	13.12	2975	14.42	4350	
	Medium	2685	25.62	5800	28.12	8485	
	Large	6420	61.26	11,850	57.45	18,270	
Hospital teaching status							<0.001
	Rural	340	3.24	1075	5.21	1415	
	Urban non-teaching	1615	15.41	3710	17.99	5325	
	Urban teaching	8525	81.35	15,840	76.80	24,365	
Admission							
	Elective	760	7.25	3735	18.11	4495	
Primary payment coverage							<0.001
	Medicare	2425	23.14	10,560	51.20	12,985	
	Medicaid	1325	12.64	2035	9.87	3360	
	Private insurance	5840	55.73	6445	31.25	12,285	
	Self-pay	520	4.96	895	4.34	1415	
	No charge	30	0.29	90	0.44	120	
	Other	330	3.15	580	2.81	910	
Median household income, $							<0.001
	1–28,999	2260	21.56	5545	26.88	7805	
	29,000–35,999	2370	22.61	5150	24.97	7520	
	36,000–46,999	2875	27.43	5380	26.08	8255	
	47,000+	2825	26.96	4220	20.46	7045	
Hospital region							<0.001
	Northeast	2110	20.13	3675	17.82	5785	
	Midwest	2675	25.52	5000	24.24	7675	
	South	3105	29.63	8110	39.32	11,215	
	West	2590	24.71	3840	18.62	6430	
Comorbidities							
	Congestive heart failure	2450	23.38	7315	35.47	9765	<0.001
	Cardiac arrhythmias	2800	26.72	8025	38.91	10,825	<0.001
	Valvular heart diseases	1085	10.35	2730	13.24	3815	<0.001
	Pulmonary circulatory disorders	395	3.77	1025	4.97	1420	0.029
	Peripheral vascular disease	830	7.92	2865	13.89	3695	<0.001
	Hypertension	6265	59.78	16,295	79.01	22,560	<0.001
	Paralysis	55	0.52	160	0.78	215	0.2602
	Other neurologic disorders	470	4.48	1425	6.91	1895	0.0002
	Chronic lung disease	1615	15.41	4125	20.00	5740	<0.001
	Diabetes mellitus	1370	13.07	6760	32.78	8130	<0.001
	Hypothyroidism	1315	12.55	2400	11.64	3715	0.3015
	CKD	730	6.97	3440	16.68	4170	<0.001
	Liver disease	320	3.05	865	4.19	1185	0.0275
	AIDS	15	0.14	60	0.29	75	0.2628
	Cancer	140	1.34	440	2.13	580	0.0261
	Rheumatologic disorders	310	2.96	555	2.69	865	0.5489
	Coagulopathy	445	4.25	1125	5.45	1570	0.0402
	Obesity	2285	21.80	4305	20.87	6590	0.4003
	Weight loss	185	1.77	490	2.38	675	0.112
	Fluid and electrolyte disorders	1770	16.89	4485	21.75	6255	<0.001
	Anemia	430	4.10	695	3.37	1125	0.1563
	Alcohol abuse	190	1.81	525	2.55	715	0.0657
	Drug abuse	400	3.82	755	3.66	1155	0.763
	Psychoses	45	0.43	50	0.24	95	0.2062
	Depression	1405	13.41	2015	9.77	3420	<0.001
	FMD	255	2.43	30	0.15	285	<0.001
	Smoking	1835	17.51	4865	23.59	6700	<0.001
	Prior MI	1225	11.69	3305	16.02	4530	<0.001
	Prior PCI	85	0.81	245	1.19	330	0.1514
	Prior CABG	300	2.86	1570	7.61	1870	<0.001
	Prior stroke	410	3.91	1420	6.88	1830	<0.001
	AMI	8000	76.34	14,555	70.57	22,555	<0.001

AIDS, acquired immunodeficiency syndrome; AMI, acute myocardial infarction; CABG, coronary artery bypass graft surgery; CKD, chronic kidney disease; FMD, fibromuscular dysplasia; MI, myocardial infarction; OMT, optimal medical therapy; PCI, percutaneous coronary intervention; SCAD, spontaneous coronary artery dissection. Variables with less than 10 in any of the cells are not reported according to Agency for Healthcare Research and Quality’s data use agreement.

Using the multivariable regression model, we found that SCAD patients who underwent PCI were associated with in-hospital mortality (odds ratio (OR) 1.89, 95% confidence interval (CI) (1.24–2.90), *p* = 0.0003), cardiogenic shock (OR 2.29, 95% CI (1.71–3.07), *p* < 0.0001), use of a left ventricular assist device (LVAD) (OR 3.97, 95% CI (2.42–6.53), *p* < 0.0001), or use of an intra-aortic balloon pump (IABP) (OR 2.24, 95% CI (1.63–3.09), *p* <0.0001). There were trends that SCAD patients who underwent PCI were associated with cardiac arrests, extracorporeal membrane oxygenation (ECMO) and development of AKI. The cost of hospitalization was higher in the PCI group (*p*-value < 0.001) and so was the length of stay (*p*-value < 0.001) (Table 2).

**Table 2.** **Outcomes of SCAD patients between PCI and OMT**.

Outcomes	OMT		PCI		*p*-value	Adjusted OR	Lower limit	Upper limit	*p*-value
	n	%	n	%					
Mortality	170	1.62	1170	5.67	<0.001	1.89	1.24	2.90	0.003
Cardiac arrest	340	3.24	895	4.34	0.04	1.12	0.78	1.61	0.521
Cardiogenic shock	400	3.82	2295	11.13	<0.001	2.29	1.71	3.07	<0.001
Use of MCS									
LVAD	105	1	1110	5.38	<0.001	3.97	2.42	6.53	<0.001
IABP	300	2.86	1855	8.99	<0.001	2.24	1.63	3.09	<0.001
ECMO	40	0.38	100	0.48	0.570	0.79	0.20	3.07	0.736
AKI	760	7.25	3085	14.96	<0.001	1.14	0.89	1.45	0.307
Cost of hospitalization, USD	16,408 ± 21,948		33,880 ± 29,774		<0.001				
Length of stay, days	3.49 ± 3.63		4.49 ± 5.75		<0.001				

AKI, acute kidney injury; ECMO, extracorporeal membrane oxygenation; IABP, intra-aortic balloon pump; LVAD, left ventricular assist device; MCS, mechanical circulatory support; OMT, optimal medical therapy; PCI, percutaneous coronary intervention; SCAD, spontaneous coronary artery dissection; OR, odds ratio. OMT as reference category.

We further conducted a secondary analysis using propensity-score matching between patients receiving PCI and OMT. Baseline characteristics of this cohort of patients are shown in Table 3. Overall, we observed a balanced cohort of 7170 pairs of SCAD patients. As a result, we measured the outcomes using two separate models. Model 1 incorporated the propensity-score matched data while Model 2 was further adjusted for the characteristics that were significant on univariate analysis. The results of both models are shown in Table 4. As observed in the maximally adjusted Model 2, the PCI cohort was associated with a higher risk of in-hospital mortality compared to the OMT cohort, but marginally missed the statistical significance threshold (OR 1.58, 95% CI (1.00–2.50), *p* = 0.05) (Table 4). Results of the other outcomes remained largely aligned with primary analysis, whereby the risk of cardiogenic shock, use of IABP and LVAD remained significantly higher in the PCI group compared to the OMT group. Table 5 showed predictor mortality of SCAD patients who underwent PCI while Table 6 showed the predicted mortality of SCAD patients who were treated with OMT.

**Table 3.** **Baseline characteristics of SCAD patients between OMT versus PCI using propensity-score matched data**.

Variables		OMT		PCI		Total	*p*-value
Number of patients, n		7170		7170		14,340	
Age		58.58 ± 14.67		57.74 ± 13.45			0.11
Female		5090	70.99	4585	63.95	9675	0.00
Race							0.82
	White	5155	71.90	5245	73.15	10,400	
	Black	1005	14.02	905	12.62	1910	
	Hispanic	645	9.00	620	8.65	1265	
	Asian or Pacific Islander	160	2.23	160	2.23	320	
	Native American	25	0.35	20	0.28	45	
	Other	180	2.51	220	3.07	400	
Hospital bed size							0.39
	Small	945	13.18	1045	14.57	1990	
	Medium	1925	26.85	1995	27.82	3920	
	Large	4300	59.97	4130	57.60	8430	
Hospital teaching status							0.78
	Rural	290	4.04	325	4.53	615	
	Urban non-teaching	1155	16.11	1180	16.46	2335	
	Urban teaching	5725	79.85	5665	79.01	11,390	
Admission							
	Elective	685	9.55	890	12.41	1575	0.02
Primary payment coverage							0.24
	Medicare	2240	31.24	2515	35.08	4755	
	Medicaid	885	12.34	830	11.58	1715	
	Private insurance	3420	47.70	3275	45.68	6695	
	Self-pay	380	5.30	315	4.39	695	
	No charge	30	0.42	15	0.21	45	
	Other	215	3.00	220	3.07	435	
Median household income, $							0.84
	1–28,999	1755	24.48	1665	23.22	3420	
	29,000–35,999	1650	23.01	1705	23.78	3355	
	36,000–46,999	2000	27.89	1980	27.62	3980	
	47,000+	1765	24.62	1820	25.38	3585	
Hospital region							0.58
	Northeast	1435	20.01	1430	19.94	2865	
	Midwest	1825	25.45	1690	23.57	3515	
	South	2365	32.98	2520	35.15	4885	
	West	1545	21.55	1530	21.34	3075	
Comorbidities							
	Congestive heart failure	1900	26.50	2095	29.22	3995	0.10
	Cardiac arrhythmias	2140	29.85	2445	34.10	4585	0.01
	Valvular heart diseases	805	11.23	875	12.20	1680	0.43
	Pulmonary circulatory disorders	335	4.67	335	4.67	670	1.00
	Peripheral vascular disease	660	9.21	790	11.02	1450	0.11
	Hypertension	4820	67.22	4915	68.55	9735	0.44
	Paralysis	55	0.77	55	0.77	110	1.00
	Other neurologic disorders	365	5.09	390	5.44	755	0.68
	Chronic lung disease	1245	17.36	1235	17.22	2480	0.92
	Diabetes	1270	17.71	1605	22.38	2875	0.00
	Hypothyroidism	880	12.27	855	11.92	1735	0.78
	CKD	645	9.00	895	12.48	1540	0.00
	Liver disease	265	3.70	275	3.84	540	0.85
	AIDS	15	0.21	15	0.21	30	1.00
	Cancer	125	1.74	145	2.02	270	0.58
	Rheumatologic disorders	230	3.21	195	2.72	425	0.44
	Coagulopathy	290	4.04	335	4.67	625	0.41
	Obesity	1630	22.73	1655	23.08	3285	0.83
	Weight loss	150	2.09	155	2.16	305	0.90
	Fluid and electrolyte disorders	1330	18.55	1355	18.90	2685	0.81
	Anemia	265	3.70	265	3.70	530	1.00
	Alcohol abuse	160	2.23	175	2.44	335	0.71
	Drug abuse	280	3.91	315	4.39	595	0.51
	Psychoses	35	0.49	25	0.35	60	0.56
	Depression	915	12.76	875	12.20	1790	0.65
	FMD	30	0.42	30	0.42	60	1.00
	Smoking	1475	20.57	1510	21.06	2985	0.75
	Prior MI	930	12.97	975	13.60	1905	0.61
	Prior PCI	65	0.91	75	1.05	140	0.69
	Prior CABG	250	3.49	445	6.21	695	0.00
	Prior stroke	350	4.88	395	5.51	745	0.63
	AMI	5350	74.62	5295	73.85	10,645	0.63

AIDS, acquired immunodeficiency syndrome; AMI, acute myocardial infarction; CABG, coronary artery bypass graft surgery; CKD, chronic kidney disease; FMD, fibromuscular dysplasia; MI, myocardial infarction; OMT, optimal medical therapy; PCI, percutaneous coronary intervention; SCAD, spontaneous coronary artery dissection.

**Table 4.** **Outcomes of SCAD patients between OMT versus PCI using propensity-score matched data**.

Outcomes	Model 1				Model 2			
	Adjusted OR	Lower limit	Upper limit	*p*-value	Adjusted OR	Lower limit	Upper limit	*p*-value
Mortality	1.87	1.19	2.92	0.006	1.58	1.00	2.50	0.051
Cardiac arrest	1.16	0.79	1.70	0.443	1.04	0.69	1.56	0.844
Cardiogenic shock	2.11	1.55	2.87	<0.001	1.91	1.39	2.62	<0.001
Use of MCS								
LVAD	3.49	1.94	6.27	<0.001	3.11	1.72	5.64	<0.001
IABP	2.36	1.69	3.31	<0.001	2.16	1.54	3.03	<0.001
ECMO	1.60	0.52	4.97	0.413	1.26	0.42	3.82	0.678
AKI	1.38	1.09	1.76	0.009	1.12	0.85	1.47	0.409

AKI, acute kidney injury; ECMO, extracorporeal membrane oxygenation; IABP, intra-aortic balloon pump; LVAD, left ventricular assist device; MCS, mechanical circulatory support; OMT, optimal medical therapy; PCI, percutaneous coronary intervention; SCAD, spontaneous coronary artery dissection; OR, odds ratio. OMT as reference category. Model 1: using propensity-score matched data. Model 2: Model 1 + adjusted for imbalance covariates including gender, elective admission, cardiac arrhythmias and chronic kidney disease.

**Table 5.** **Predicted mortality of SCAD patients who underwent PCI**.

Variables	Odds ratio	Lower limit	Upper limit	*p*-value
Cardiogenic shock	10.14	6.86	14.99	<0.001
Age	1.05	1.03	1.06	<0.001
Female	1.59	1.15	2.18	0.005
Smoking	0.89	0.58	1.37	0.600
Fluid and electrolyte disorders	1.68	1.19	2.36	0.003
Weight loss	1.15	0.50	2.66	0.740
Obesity	0.65	0.43	1.00	0.050
Coagulopathy	1.34	0.77	2.31	0.298
Liver disease	0.88	0.60	1.30	0.519
CKD	1.75	1.01	3.03	0.045
Other neurological disorders	2.24	1.45	3.47	<0.001
Diabetes mellitus	1.82	1.31	2.53	<0.001
Peripheral vascular disease	1.24	0.85	1.80	0.272
Pulmonary circulatory disorders	1.16	0.67	2.00	0.598
Valvular heart diseases	0.78	0.51	1.20	0.265
Cardiac arrhythmia	1.52	1.09	2.10	0.013
Congestive heart failure	1.13	0.81	1.57	0.483
Primary payment coverage				
Medicare	Ref			
Medicaid	0.67	0.32	1.41	0.290
Private insurance	0.82	0.51	1.33	0.428
Self-pay	0.97	0.37	2.60	0.959
No charge	N/A	N/A	N/A	N/A
Other	0.88	0.28	2.72	0.823

CKD, chronic kidney disease; PCI, percutaneous coronary intervention; SCAD, spontaneous coronary artery dissection. Variables with less than 10 in any of the cells are not reported according to Agency for Healthcare Research and Quality’s data use agreement and are marked as N/A.

**Table 6.** **Predicted mortality of SCAD patients who were treated with OMT**.

Variables	Odds ratio	Lower limit	Upper limit	*p*-value
Cardiogenic shock	8.96	2.86	28.07	<0.001
Age	1.07	1.04	1.11	<0.001
Female	0.50	0.22	1.17	0.111
Fluid and electrolyte disorders	2.65	1.06	6.59	0.036
Weight loss	1.36	0.33	5.54	0.672
Rheumatologic disorders	3.32	0.71	15.48	0.126
Liver disease	1.73	0.68	4.40	0.249
CKD	1.22	0.30	5.03	0.778
Other neurological disorders	5.68	1.78	18.15	0.003
Pulmonary circulatory disorders	1.27	0.43	3.73	0.659
Peripheral vascular disease	2.18	0.45	10.52	0.330
Cardiac arrhythmia	1.25	0.50	3.12	0.637
Congestive heart failure	0.47	0.19	1.18	0.107
Elective admission	1.68	0.55	5.19	0.365
Primary payment coverage				
Medicare	Ref			
Medicaid	2.96	0.70	12.51	0.140
Private insurance	0.71	0.21	2.42	0.580
Self-pay	3.65	0.65	20.60	0.143
No charge	N/A	N/A	N/A	N/A
Other	N/A	N/A	N/A	N/A

CKD, chronic kidney disease; OMT, optimal medical therapy; SCAD, spontaneous coronary artery dissection. Variables with less than 10 in any of the cells are not reported according to Agency for Healthcare Research and Quality’s data use agreement and are marked as N/A.

## 4. Discussion

In our national study, there were 3 main findings. First, the temporal trend of PCI and OMT among SCAD had shifted, with a yearly decrease in the percentage of patients receiving PCI, for the years 2016–2020. This is likely due to more data on SCAD management leaning towards medical therapy and a more conservative approach. Most importantly, both the American Heart Association (AHA) scientific statement and the European society of cardiology Expert opinion recommend conservative management of SCAD in stable cases, as SCAD is known to heal with the resorption of intramural hematoma overtime unlike ischemia secondary to atherosclerotic plaque [7, 9]. This recommendation is consistent with our prior meta-analysis, which showed no difference in terms of long-term mortality and recurrent SCAD among patients with SCAD treated with medical therapy compared with those treated with PCI [10].

Second, we found SCAD patients who underwent PCI were associated with in-hospital mortality, cardiogenic shock, LVAD, and IABP. This finding suggests that the baseline of SCAD patients who underwent PCI were much sicker compared to SCAD patients who were treated with medical therapy. SCAD patients with comorbidities (e.g., hypertension, diabetes mellitus, CKD, heart failure, shock) may be considered as a high-risk SCAD phenotype and may require intervention rather than conservative management. PCI and medical management have both been used in both case series and retrospective studies looking at SCAD management in inpatients [11, 12, 13]. The choice of which management to choose has been guided in these cases by the degree of coronary artery obstruction, severity of symptoms at presentation, whether the patient has acute coronary syndrome at presentation or not, and their coronary artery anatomy. SCAD patients with comorbidity or high-risk features probably underwent PCI rather than medical therapy.

Third, the mortality predictors of SCAD patients who underwent PCI were cardiac arrhythmia or acutely decompensated heart failure. SCAD patients with ventricular arrhythmia are likely to get treated with PCI rather than medical therapy and mortality is higher. There are technical challenges for PCI and SCAD patients. A study has reported variable success rates, with PCI success rates reports ranging from 29 to 92% [12]. With procedural failure and recurrence, a possibility. There are some potential risks to having PCI during SCAD, and these are thought to be the drivers of the failure rates. These risks include possible iatrogenic secondary dissection, where the guide wire engages with the false lumen which is then enlarged during ballon dilation [12].

As the intervention is offered based on clinical decision and patient presentation, patients with SCAD and other co-morbidities may present initially more unstable with vital sign or laboratory abnormalities, and need urgent intervention, such as cardiac catheterization, which leads to PCI placement. However, this is not yet clearly understood in the literature. There is no data from RCTs comparing medical therapy and PCI. We previously discussed that revascularization is associated with suboptimal procedural success rates and high rates of complications despite preserved coronary flow [14]. Long term follow up is recommended to ensure management is working, and that further interventions are not necessary for symptom management. More research is needed to understand optimal interventional guidelines and medical management to be implemented.

The current study has certain limitations that should be taken into consideration while interpreting the results. The major limitation was inherent to the database itself. While the NIS database has a strength in its huge sample size and ability to extrapolate to the US population, the lack of detailed clinical information, such as specific indications for PCI and comprehensive angiographic findings, including coronary flow, limits our ability to assess procedural outcomes and patient selection fully. Furthermore, key clinical data, such as patient presentation, laboratory results, imaging or echocardiographic findings, and medication use before, during, and after SCAD diagnosis, were not readily available within the database.

## 5. Conclusions

In this retrospective study looking at the NIS data base over four years we saw SCAD patients who underwent PCI are likely to be much sicker and have more comorbidities and higher rate of mortality, compared to SCAD patients who were treated with medical therapy. SCAD patients with heart failure and ventricular arrhythmia who underwent PCI were associated with higher mortality.

## Availability of Data and Materials

The data sets generated and/or analyzed during the current study are not publicly available due to HCUP data policy but are available from the corresponding author on reasonable request.

## References

[1] Tweet MS, Gulati R, Hayes SN (2016). Spontaneous Coronary Artery Dissection. *Current Cardiology Reports*.

[2] Hayes SN, Tweet MS, Adlam D, Kim ESH, Gulati R, Price JE (2020). Spontaneous Coronary Artery Dissection: JACC State-of-the-Art Review. *Journal of the American College of Cardiology*.

[3] Kok SN, Hayes SN, Cutrer FM, Raphael CE, Gulati R, Best PJM (2018). Prevalence and Clinical Factors of Migraine in Patients With Spontaneous Coronary Artery Dissection. *Journal of the American Heart Association*.

[4] Di Fusco SA, Rossini R, Zilio F, Pollarolo L, di Uccio FS, Iorio A (2022). Spontaneous coronary artery dissection: Overview of pathophysiology. *Trends in Cardiovascular Medicine*.

[5] Waterbury TM, Tweet MS, Hayes SN, Eleid MF, Bell MR, Lerman A (2018). Early Natural History of Spontaneous Coronary Artery Dissection. *Circulation. Cardiovascular Interventions*.

[6] Waterbury TM, Tarantini G, Vogel B, Mehran R, Gersh BJ, Gulati R (2020). Non-atherosclerotic causes of acute coronary syndromes. *Nature Reviews. Cardiology*.

[7] Hayes SN, Kim ESH, Saw J, Adlam D, Arslanian-Engoren C, Economy KE (2018). Spontaneous Coronary Artery Dissection: Current State of the Science: A Scientific Statement From the American Heart Association. *Circulation*.

[8] Tweet MS, Eleid MF, Best PJM, Lennon RJ, Lerman A, Rihal CS (2014). Spontaneous coronary artery dissection: revascularization versus conservative therapy. *Circulation. Cardiovascular Interventions*.

[9] Adlam D, Alfonso F, Maas A, Vrints C, Writing Committee (2018). European Society of Cardiology, acute cardiovascular care association, SCAD study group: a position paper on spontaneous coronary artery dissection. *European Heart Journal*.

[10] Krittanawong C, Nazir S, Hassan Virk H, Hahn J, Wang Z, Fogg SE (2021). Long-Term Outcomes Comparing Medical Therapy versus Revascularization for Spontaneous Coronary Artery Dissection. *The American Journal of Medicine*.

[11] Maeder M, Ammann P, Angehrn W, Rickli H (2005). Idiopathic spontaneous coronary artery dissection: incidence, diagnosis and treatment. *International Journal of Cardiology*.

[12] Velagapudi P, Kirtane AJ, Saw J (2023). Spontaneous Coronary Artery Dissection Causing Acute Myocardial Infarction: Is Revascularization the Best Course of Action?. *JACC. Cardiovascular Interventions*.

[13] Al Emam ARA, Almomani A, Gilani SA, Khalife WI (2016). Spontaneous Coronary Artery Dissection: One Disease, Variable Presentations, and Different Management Approaches. *The International Journal of Angiology: Official Publication of the International College of Angiology, Inc*.

[14] Krittanawong C, Gulati R, Eitzman D, Jneid H (2021). Revascularization in Patients With Spontaneous Coronary Artery Dissection: Where Are We Now?. *Journal of the American Heart Association*.

